# Effects of High Hydrostatic Pressure Combined with Vacuum-Freeze Drying on the Aroma-Active Compounds in Blended Pumpkin, Mango, and Jujube Juice

**DOI:** 10.3390/foods10123151

**Published:** 2021-12-20

**Authors:** Lin Yuan, Xujuan Liang, Xin Pan, Fei Lao, Yong Shi, Jihong Wu

**Affiliations:** 1 College of Food Science and Nutritional Engineering, China Agricultural University, Beijing 100083, China; yuanlin@cau.edu.cn (L.Y.); 2018306100327@cau.edu.cn (X.L.); xinpan.cau@gmail.com (X.P.); fei.lao@cau.edu.cn (F.L.); 2 National Engineering Research Center for Fruit and Vegetable Processing, Beijing 100083, China; 3 Key Laboratory of Fruit and Vegetable Processing, Ministry of Agriculture and Rural Affairs, Beijing 100083, China; 4 Beijing Key Laboratory for Food Non-Thermal Processing, Beijing 100083, China; 5 Haoxiangni Health Food Co., Ltd., Xinzheng 451100, China; shiyongzzxz@126.com

**Keywords:** high hydrostatic pressure, vacuum freeze drying, aroma compounds, blended juice, gas chromatography–olfactometry

## Abstract

A combination process of completely non-thermal processing methods involving high hydrostatic pressure (HHP) and vacuum-freeze drying (VFD) for producing a new snack from fruit and vegetable blends was developed, and the effect of the process on flavor quality was investigated. The HHP–VFD treatment did not significantly reduce volatile compound contents compared to single HHP or VFD. Gas chromatography–olfactometry showed that HHP–VFD raised the contents of floral-like volatile compounds (e.g., β-ionone) compared to the untreated sample. Sensory evaluation analysis confirmed that the overall liking was unchanged after the HHP–VFD treatment. The HHP–VFD combined treatment is effective in maintaining the flavor and extending shelf life, and is convenient for the portability and transportation of ready-to-drink juice.

## 1. Introduction

Flavor quality is a composite of oronasal sensory responses to aromas and taste, and is the main attribute controlling consumers’ choices of fruit and vegetable juices [1]. Ready-to-drink (RTD) fruit and vegetable juices are increasingly preferred by consumers since they have similar fresh flavors without inconvenient operations (e.g., washing, peeling, or cutting) compared with fresh fruits and vegetables. However, the extremely short shelf life and low circulation due to unsterilized processing limit the RTD juices to be sold only in stores, restaurants, and bars. Therefore, the effective sterilization of RTD fruit and vegetable juices is required, but traditional thermal pasteurization can cause a pronounced decrease in flavor quality in juices [2,3].

High hydrostatic pressure (HHP) is becoming a first-choice sterilization method to fulfill the requirements of nonthermal processing, since its limited effects on covalent bonds improve the retention of flavor quality in comparison with thermal processes [4,5]. As reported, HHP-treated mango juice [6], cloudy apple juice [7], and cucumber juice [8] retain much better flavor quality and are closer to fresh juices than pasteurized juices. However, HHP-processed juices may have a limited shelf life due to residual enzyme activity that can undesirably alter flavor during storage. For example, a previous study on the shelf life of HHP-treated and pasteurized orange juice showed that after 8 weeks of storage at 4 °C, the sensory quality and major volatiles content of HHP-treated orange juice were lower than those of pasteurized orange juice [9]. Thus, it is urgent to explore new processing methods to improve the shelf life of HHP-treated fruit and vegetable juices. HHP is often combined with other technologies such as gas, ultrasound, and ultrafiltration to improve safety and quality [10,11,12].

Vacuum-freeze drying (VFD) is extensively employed as one non-thermal drying tool because VFD almost reserves the same color, flavor, and nutrition as fresh fruits and vegetables [13,14]. VFD is confirmed to significantly prolong the shelf life of fruits and vegetables, such as strawberries [15] and jujubes [16]. In recent years, new types of snacks (bars, tablets, cubes) made from fruits and vegetables by VFD have become popular worldwide because of their long shelf life, portability, convenience, good taste, and nutrition [17]. Therefore, combining HHP with VFD is a new method to make freeze-dried fruit and vegetable products with high flavor quality and long shelf life. Research on the nutritional effects of HHP–VFD is widely reported [18,19], but to the best of our knowledge, there has been no study on the effect of HHP combined with VFD on food flavor quality.

Herein, three common fruit and vegetable raw materials in China, including pumpkin, mango, and jujube, were selected. The study aimed to (1) identify and quantify volatiles through headspace solid-phase microextraction (HS-SPME) with gas chromatography–mass spectrometry (GC–MS); (2) discriminate the major aroma-active compounds with detection frequency and odor activity value analyses; (3) characterize the changes in volatiles; (4) validate the sensory differences by quantitative descriptive analysis, in the fruit/vegetable blended juices treated by HHP or HHP–VFD.

## 2. Materials and Methods

### 2.1. Raw Materials and Chemicals

Jujubes (*Ziziphus jujuba* Mill. cv. Hetian), pumpkins (*Cucurbita moschata* Duch. cv. Beibei), and mangoes (*Mangifera indica* L. cv. Qingmang) without physical injuries or disease infection were purchased from a local retail market in January 2021 in Beijing, China.

*N*-Alkanes (C_7_-C_30_) for qualitative tests were bought from Sigma (St. Louis, MO, USA). The internal standard, 2-octanol, for quantitative tests was bought from Aladdin Biochemical (Shanghai, China). Other analytical reagents (e.g., NaCl) were made by Beijing Chemical Reagent Company (Beijing, China).

### 2.2. Juices Preparation

Juices were prepared as per Xu et al. [20] with some modifications. After preliminary soaking in water for 10 min and removing the cores, the jujubes were pulped in a bench-scale juicer (Joyong Co., Ltd., Qingdao, China) with a solid/water ratio of 1:3 and then filtered by a nylon cloth filter. The pumpkins and mangoes were both pulped with the solid/liquid ratio of 1:3 after removing cores and peels, and filtered through 60 meshes. These three types of juices were blended with the liquid ratio of jujube juice/pumpkin juice/mango juice of 1:2:2, according to a sensory and orthogonal test performed by Haoxiangni Health Food Co., Ltd. (Xinzheng, China). The blended juices were placed in 100 mL and 0.065 cm-thick polyethylene terephthalate bottles (Yixiubogu e-commerce platform, Beijing, China). The total sugar content of the blended juices was determined to be 286.19 ± 5.00 mg/g using anthrone–sulfuric acid colorimetry [21], and the pH was 4.95 ± 0.05.

### 2.3. HHP of the Blended Juices

**Figure 1:**
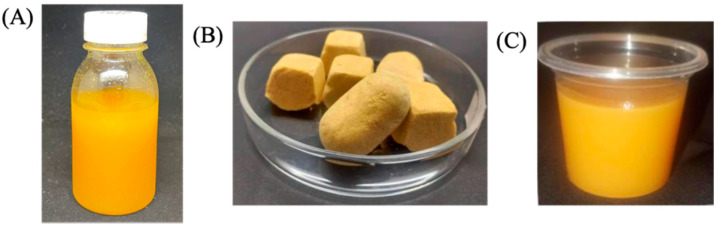
Appearance of HHP-treated blended juices and HHP–VFD-treated blended cubes. (**A**) HHP3 sample (blended juice treated with HHP at 600 MPa). (**B**) HHP3–VFD sample (blended cube of HHP3 juice treated after VFD). (**C**) Rehydrated HHP3–VFD juice.

To carry out HHP, fresh blended juices in 100 mL bottles were pressurized using a CQC30L-600 HHP pressurization unit (Suyuanzhongtian Scientific Co., Ltd., Beijing, China) as per Zhang et al. [22]. Pressurization was operated with distilled water as the transmitting fluid and at a rate of ~200 MPa/min. The bottles were treated at 200 (HHP1), 400 (HHP2), and 600 MPa (HHP3) for 10 min at 25 °C, followed immediately by decompression to minimize adiabatic heating. The fresh blended juices were taken as the control. Samples of HHP1, HHP2, some fresh and HHP3 juices (Figure 1A) were frozen in liquid nitrogen instantly and kept at −80 °C until used within two weeks. Other fresh and HHP3 juices were prepared for VFD.

### 2.4. VFD of the Blended Cubes

After preliminary freezing at −20 °C for 24 h, each cell for 5 g of fresh or HHP3-treated blended juices was injected into silastic molds (Haoxiangni Health Food Co., Ltd., Xinzheng, China). VFD was run at −40 °C (cold trap) and 100 Pa (absolute pressure) for 48 h on an LGJ-25C freeze dryer (Foring Technology Development Co., Ltd., Beijing, China). The products were named VFD and HHP–VFD blended cubes (Figure 1B), respectively. The moisture of the blended VFD cubes was 8.50 ± 0.50% (wet basis), which is a safe level for long-term storage at room temperature [23]. After VFD, the samples were immediately placed into low-density polyethylene bags, heat-sealed, and stored in an allochroic silica gel dryer at room temperature.

### 2.5. Determination of Color Parameters

Colors of the samples were measured at ambient temperature using a color difference meter (ColorQuest XE, Hunter Associated Laboratory Inc., Reston, VA, USA) in the transmission (for juices) or reflectance (for cubes) mode immediately after HHP or VFD. The meter was calibrated using a white color standard before measurements. Colors were recorded in units of L*, a*, and b* uniform color space, where L* means lightness, a* ranges from negative values for green to positive values for red, and b* ranges from negative values for blue to positive values for yellow. Numerical ΔE (total color difference), C* (chroma), and h^0^ (hue angle) were calculated as follows:

(1) $$\begin{array}{c} \Delta E=\sqrt[{2}]{{\left(L^{*}-L^{*}{}_{0}\right)}^{2}+{\left(a^{*}-a^{*}{}_{0}\right)}^{2}+{\left(b^{*}-b^{*}{}_{0}\right)}^{2}} \end{array}$$

(2) $$\begin{array}{c} C^{*}=\sqrt[{2}]{{\left(a^{*}\right)}^{2}+{\left(b^{*}\right)}^{2}} \end{array}$$

(3) $$\begin{array}{c} h^{0}=\mathrm{arc}\tan(b^{*}/a^{*}) \end{array}$$

where L*_0_, a*_0_, and b*_0_ were the control values for fresh blended juice (for juices) or VFD cubes without HHP (for cubes).

### 2.6. Microstructure Analysis

The microstructure of the VFD- or HHP–VFD-treated blended cubes was observed by a field emission scanning electron microscope (SU-8020, Hitachi, Tokyo, Japan), under 100× and 500× magnification.

### 2.7. Analysis of Volatile Compounds

#### 2.7.1. Isolation

The volatile compounds were extracted using SPME according to Pang et al. [24] with minor modification. Rehydrated juices were from the HHP–VFD-treated blended cubes dissolved with aseptic water by water loss rate after VFD. At each time, the blended juice or rehydrated juice (5 g) was transferred into a 20 mL headspace bottle (ANPEL Laboratory Technologies Inc., Shanghai, China) containing 1.5 g of NaCl and 10 μL of 8.22 μg/mL 2-octanol as the internal standard. The bottle was sealed by PTFE-silicone septum and balanced under agitation at 45 °C for 10 min. Next, a 50/30 μm divinylbenzene/carboxen^TM^/polydimethylsiloxane SPME fiber (Supelco, Bellefonte, PA, USA) was exposed to the headspace of the juices for 40 min at 45 °C without agitation. Finally, the fiber was removed and placed into the GC injector at 250 °C for 5 min.

#### 2.7.2. GC–MS

An Agilent 7890 GC system coupled with an Agilent 5975C MS meter (Agilent Technologies, Santa Clara, CA, USA) was operated as per a reported method [25] with minor modification. The volatile compounds were isolated with a DB-Wax fused silica capillary column (30 m × 0.25 mm i.d. × 0.25 μm; Agilent). Helium (≥99.999%) as the carrier gas was flowed at a rate of 1.0 mL/min. The oven was kept at 35 °C for 5 min, and ramped first at the rate of 4 °C/min to 120 °C, and then to 225 °C at the rate of 5 °C/min for 5 min. MS was carried out at an electron impact mode of 70 eV and scanned within 35–550 *m*/*z*.

#### 2.7.3. Identification and Quantification

The volatile compounds in blended juices and rehydrated juices were identified by comparing the mass spectra with the NIST10 database, and by comparing the calculated linear retention index (LRI) with the open data of the NIST WebBook. LRI was computed using the retention time of n-alkanes (Sigma-Aldrich, St. Louis, MO, USA) detected under the same GC–MS temperature program. A difference between the calculated LRI and those from published data or the LRI and Odour Database (http://www.odour.org.uk/ (accessed on 14 September 2021)) less than 20 is acceptable. The concrete equation is:

(4) $$\begin{array}{c} \mathrm{LRI}=100N+\frac{100n\left(t_{\mathrm{Ra}}-t_{\mathrm{Rn}}\right)}{t_{R\left(N+n\right)}-t_{\mathrm{Rn}}} \end{array}$$

where N is the number of carbon atoms of n-alkanes on the left side of the compound, a is the difference in the number of carbon atoms of n-alkanes on both sides, t_Ra_ is the retention time of the compound, t_Rn_ and t_R(N+n)_ are the retention time of n-alkanes on the left and right sides of the compound, respectively.

The volatile compounds in blended juices and rehydrated juices were quantified. Peak areas were normalized with 2-octanol, the internal standard, added to each sample. Concentration of each detected compound was computed from the peak area ratio of its own to 2-octanol.

### 2.8. Gas Chromatography–Olfactometry (GC–O)

The aroma-active compounds were characterized using an olfactory detector port (ODP 3; Gerstel GmbH & Co. KG, Mülheim an der Ruhr, Germany) linked to the GC–MS system. After the volatile compounds were isolated by the capillary column, the same volume of the effluent was removed to both the sniffing port and the MS meter. In the GC–O sniffing port, the transfer line was kept at 230 °C and moist air was continually added at 60 mL/min to maintain the sniffing sensitivity. The same temperature program for GC–MS was adopted. Detection frequency (DF) was analyzed by four trained panelists with good and stable sensation, and each panelist repeated twice. The retention time and odor descriptions were recorded, and the odorant with DF ≥ 6 was regarded a potent aroma-active compound in a total of eight tests [26].

### 2.9. Odor Activity Value (OAV)

The OAV was calculated as follows:

(5) $$\begin{array}{c} \mathrm{OAV}=\frac{C_{i}}{{\mathrm{OT}}_{i}} \end{array}$$

where Ci is the odor concentration (detected in Section 2.7) and OTi is the threshold in water (obtained from data in the literature). A compound with OAV ≥ 1 was accepted as a potential contributor to the corresponding aroma profile [27].

### 2.10. Sensory Evaluation

According to relevant requirements of Quantitative Descriptive Analysis^®^ (QDA) [28,29], the sensory evaluation team consisted of 12 healthy nonsmoking judges were recruited from China Agricultural University (five males and seven females, aged 23–29) with rich experience in this field. During the evaluations at room temperature, 20 mL of blended juice or rehydrated juice of each sample described in Section 2.7.1 was poured into a 50 mL white paper cup (Yixiubogu e-commerce platform, Beijing, China) and randomly coded with a 3-digit number. The judges described as many sensory attributes of each sample as possible. After discussion, six sensory attributes were chosen (sweet, sour, floral, rosin, grassy, fruity). The evaluation score was a 10-point scale from 0 (not perceivable) to 9 (strongly perceivable). The average score of each attribute computed among the 12 judges was illustrated in a spider diagram.

### 2.11. Statistical Analysis

Data are expressed as mean ± standard deviation of three repetitions. Differences between samples at the significance level *p* < 0.05 were detected via one-way and two-way analysis of variance (ANOVA) and on SPSS 25.0 (SPSS Inc., Chicago, IL, USA). Differences in volatile profiles among the samples were compared by principal component analysis (PCA) and cluster analysis with MetaboAnalyst online tools (http://www.metaboanalyst.ca/ (accessed on 10 October 2021)). Plotting was finished on Graphpad prism 8.0 (Graphpad Software, San Diego, CA, USA) and Origin 2019 (OriginLab, Northampton, MA, USA).

## 3. Results and Discussion

### 3.1. Effects of HHP and VFD on Color of Blended Juices

Table 1 showed the changes in L*, a*, b*, C*, h^0^, and ΔE of HHP-treated or HHP–VFD-treated blended juices. No significant differences in color parameters of the blended juice samples were observed at all pressures of HHP (*p* > 0.05), which was consistent with previous research in juices where HHP was shown to better preserve the color of raw materials [30]. On the contrary, the values of all color parameters significantly increased (*p* < 0.05) after rehydrating from VFD-treated cubes compared to fresh blended juice, indicating a brighter and more saturated color after VFD and rehydration. VFD has been reported to retain the color characteristics of raw materials relative to other drying methods [31,32,33]. These results are consistent with the reported increases in L*, a*, or b* after rehydration from VFD of potato slices [34]. This may be due to the fact that after VFD, the water in the raw materials is replaced by air, which changes the original structure, thus leading to changed light transmission after rehydration and causing changes in color and brightness. In addition, the color parameters of VFD cubes were measured and again the HHP did not cause a large change in the color of solid.

### 3.2. Effect of HHP on Microstructure

**Figure 2:**
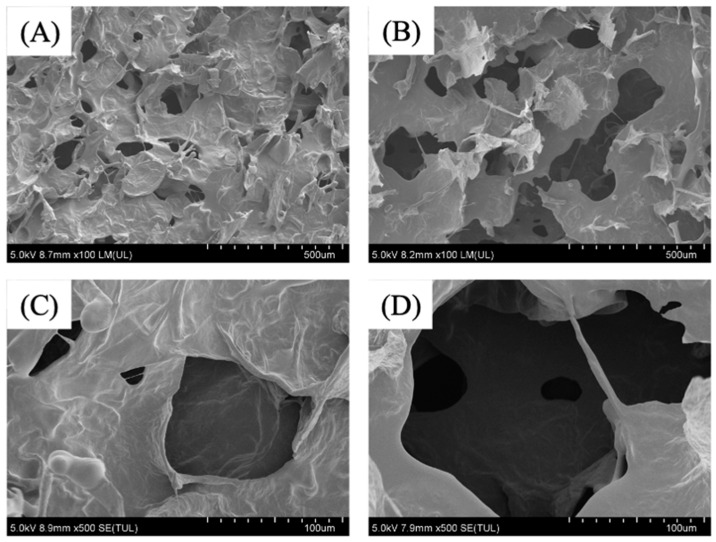
Microstructure of blended cubes. (**A**) VFD sample under 100× magnification (blended cube of fresh juice treated after VFD); (**B**) HHP3–VFD sample under 100× magnification (HHP3–VFD, blended cube of HHP3 juice treated after VFD); (**C**) VFD sample under 500× magnification (blended cube of fresh juice treated after VFD); (**D**) HHP3–VFD sample under 500× magnification (HHP3–VFD, blended cube of HHP3 juice treated after VFD).

Microstructure has an important effect on the quality of dried food. The microstructure of VFD-treated cubes changed after HHP treatment (Figure 2). Irregular and sharp pores can be observed in the VFD-treated cube (Figure 2A,C). These pores provided space for water evaporation through heat and mass transfer during VFD. After HHP, the average porosity of the microstructure increased in the HHP–VFD-treated cube (Figure 2B,D). The increase in pore size caused by HHP may be due to the degradation of cell wall structure and the enhancement on cell permeability, which contributed to the increased mobility and mass transfer rate of water. This result is consistent with some studies on high-pressure-assisted osmotic dehydrated ginger and aloe vera [35,36].

### 3.3. Effect of HHP and VFD on the Volatile Components of Blended Juices

A total of 64 volatile compounds were detected in blended juices and rehydrated juices, including 10 ketones, 19 aldehydes, 8 terpenes, 8 alcohols, 9 acids, and 10 esters (Table 2). Most of the volatiles found here are the same as in other reports on jujube, pumpkin, mango fruits, or their products [37,38,39,40,41], except for two compounds: 1-(1,3-dimethyl-3-cyclohexen-1-yl) ethanone (A4 in Table 2) and 5-ethyl-1-cyclopentene-1-carboxaldehyde (B12 in Table 2). Hence, appropriate blending can improve the sensory qualities and increase kinds of volatile compounds [42,43].

Hydrocarbon aldehydes (e.g., hexanal, (*E*)-2-hexenal, benzaldehyde), terpenes (e.g., 3-carene), acids (e.g., hexanoic acid, heptanoic acid, octanoic acid), esters (e.g., ethyl acetate, methyl butyrate), and some other ketones, alcohols, and benzene derivatives were predominant in the volatile compounds of blended juices and rehydrated juices. As reported, HHP can alter the compositions and quantities of volatile compounds in many fruit and vegetable juices and wines [44,45,46]. The reason may be that HHP alters the weak chemical bonds of enzymes, thus modifying the secondary to quaternary structures of key enzymes in the production of aroma compounds [1]. In general, the VFD process results in a decrease in volatile compounds. Such decreases in freeze-dried apple reportedly occurred mainly during ice sublimation, where volatile compounds with higher vapor pressure than water molecules were excluded and evaporated when the sample matrix exceeded its glass transition temperature [47]. In comparison, the decrease in freeze-dried durian pulp may occur during sample preparation before drying and grinding into powder [48]. However, compared with other drying methods (e.g., hot air drying, solar drying and heat-pump drying), VFD shows a better retention in volatile compounds [49]. Herein, VFD caused a reduction in volatile compounds in rehydrated juices by 56.7% to 63.5%, compared to fresh blended juice.

#### 3.3.1. Ketones

**Figure 3:**
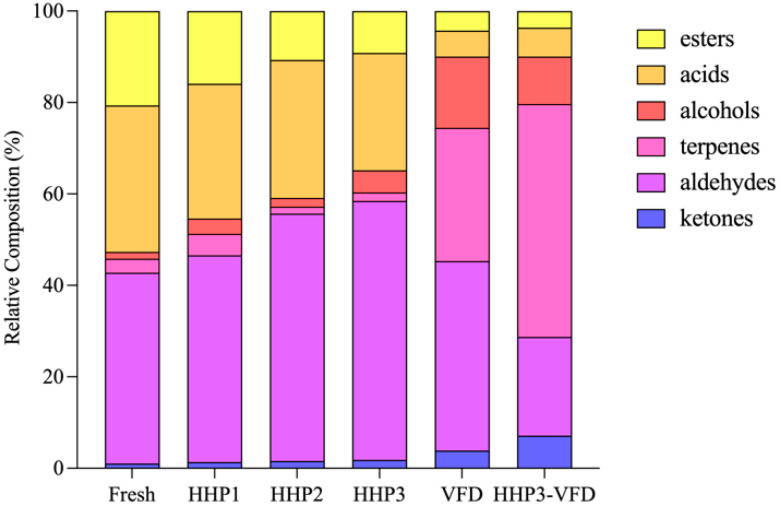
Relative composition (%) of classes of volatile components of blended juice treated by HHP and VFD.

Ketones are important components of fruit and vegetable flavor profiles, although most of them possess a high odor threshold compared to aldehydes or esters [50]. In total, 10 ketones were detected in all six samples. The contents of acetone and acetoin in HHP-treated blended juices decreased (*p* < 0.05) with the pressure of HHP. Pressure levels of HHP may enhance or retard enzymatic and chemical reactions and thereby indirectly influence the increase or decrease in volatile compounds [5]. As reported, the concentrations decreased in some ketones, while increased in some others under different pressures in green asparagus juice [45]. Compared to fresh blended juice, HHP slightly decreased (*p* < 0.05) the contents of total ketones, whereas HHP–VFD led to a twofold rise (*p* < 0.05). In addition, the proportion of total ketones was low in HHP-treated blended juices and was less than 2% of total volatiles, but was higher in HHP–VFD-treated rehydrated juices (Figure 3). This result is in agreement with a study on yogurt melts that the amounts of ketones increased after freeze drying [51]. 2-Butanone and β-damascenone were only found in HHP-treated samples, and geranylacetone and β-ionone were only detected in VFD-treated samples. This result implies that these components are exclusive to processed juice and resulted from reactions occurring during processing. 1-(1,3-Dimethyl-3-cyclohexen-1-yl) ethanone was found in the essential oils of some genus *Nepeta* plants [52], and as we know, was identified in blended juice for the first time, though it was not the major aroma contributor.

#### 3.3.2. Aldehydes

Nineteen aldehydes were detected, which were the most representative volatile compounds in blended juices. Most of these aldehydes belong to C6–C9 aldehydes, which originate from the oxidative breakdown and cleavage of fatty acids by lipoxygenase (LOX) and hydroperoxide lyase (HPL) [53]. The hexanal content of HHP-treated blended juices increased (*p* < 0.05) from 22.66 μg/kg in fresh blended juice to 77.88 μg/kg at 400 MPa and 102.59 μg/kg at 600 MPa. This result is similar to a previous study on the effect of HHP on kiwifruit pulp beverages [54]. HPL was in direct charge for the generation of hexanal [55], and was not completely inactivated at pressures below 600 MPa [56]. On the contrary, the contents of other aldehydes such as benzaldehyde, (*E*)-2-nonenal, and (*E*,*Z*)-2,6-nonadienal in HHP-treated blended juice significantly declined (*p* < 0.05) in comparison with fresh blended juice. These aldehydes resulted from the peroxidation of polyunsaturated fatty acids under catalysis by LOX, which were completely inactivated in tomatoes at 550 MPa [56]. Similarly, these aldehydes decreased in Keitt mango juice [6] and mulberry juice [57] after HHP. HHP–VFD increased (*p* < 0.05) the contents of (*E*, *E*)-2,4-heptadienal and β-cyclocitral, which may be due to the decomposition of unsaturated fatty acids during drying. Linolenic acid degradation leads to the formation of 2,4-heptadienal isomers, which is the same as in mango peels [53,58]. β-Cyclocitral originates from the degradation of carotenoids, and may be used as a differentiator compound between VFD-treated and non-VFD-treated samples.

#### 3.3.3. Terpenes

Terpenes account for the typical aroma profiles of many fruits, particularly tropical fruits such as mangoes [59]. One of the most ubiquitous terpenes is limonene, but it is not a powerful odorant. HHP decreased (*p* < 0.05) the content of total terpenes in blended juices, which is similar to the results in different varieties of orange juices [44,60]. Interestingly, while HHP–VFD increased (*p* < 0.05) the content of total terpenes, some new terpenes appeared (e.g., α-phellandrene, myrcene, DL-limonene, β-thujene, α, p-dimethylstyrene), which is different from previous studies on freeze-dried cabbages and carrot slices [61,62]. This difference may be caused by variations in substrate, pressure, and pretreatment conditions. VFD did not necessarily result in the inactivation of some cold-resistant enzymes, which may be inactivated by high pressure during HHP at room temperature. Another possible reason was the breaking of the cells in which the terpenes were stored during VFD, as some sesquiterpenes reportedly increased in freeze-dried laurel leaves [63]. Additionally, de Torres et al. [64] observed an increase in the amount of norisoprenoids in freeze-dried grape skins, a fact that is attributed to the rise of hydrolysis of glycosides, which will cause the release of aglycones, resulting from the decrease in water and increase in acidity during VFD.

#### 3.3.4. Alcohols

In total, eight alcohols were identified, which all possessed only one hydroxyl group. The proportion of total alcohols rose after processing (Figure 3), which mainly derived from many oxidative hydration–dehydration reactions of hydrocarbon terpenes and other precursors under juice acidic conditions [65]. Similarly, total alcohols increased in HHP-treated orange juices and freeze-dried grape skins [44,64]. The 1-octen-3-ol content significantly increased (*p* < 0.05) after HHP treatments. Moreover, the level of 1-octen-3-ol in HHP-treated kiwifruit pulp beverage and black truffle significantly increased or appeared after the treatment [54,66]. This result may be interpreted as the activation of some glycosidases by HHP, which released the glycoside-bound alcohol in blended juices [1]. Five kinds of alcohols were detected only in HHP–VFD-treated rehydrated juices, of which carveol was a terpene alcohol that contributed a woody turpeny note to mango puree [67]. Carveol was formed from the auto-oxidation of limonene, which is consistent with limonene detected only in HHP–VFD-treated samples. p-Cymen-8-ol is another abundant aromatic compound reported in the pulp of ripe mango fruits [68], and is considered to be derived from γ-terpinene, which was biosynthesized under the influence of oxygen. The appearance of p-cymen-8-ol is in agreement with a previous study on fresh and freeze-dried carrots [62].

#### 3.3.5. Acids

Nine acids were detected in blended juices, which occupied the second-largest proportion of all volatile compounds. The content of all acids in the samples decreased (*p* < 0.05) after HHP treatment. However, this decrease was not linear with a high-pressure increase. The most acid retention was observed for blended juices pressure treated at 400 MPa, which is consistent with the study of Zabetakis et al. [69] on the effects of HHP on butanoic acid, 2-methyl-butanoic acid, and hexanoic acid in strawberries. Since these short- and medium-chain fatty acids, or volatile fatty acids, are generally described as unpleasant odors as stale or pungent, HHP can significantly improve the perception of odor and flavor [70]. Only five acids existed in HHP–VFD-treated rehydrated juices, and their contents were significantly lower (*p* < 0.05) than in the juices. As reported, acids disappeared and decreased after freeze drying in jujubes, which was much more pronounced than other dehydration methods such as air drying and microwave drying, which evidenced the improvement in flavor by freeze drying [71].

#### 3.3.6. Esters

Esters are pivotal to the aroma of most fruits and comprise the important proportion of volatiles in jujubes and mangoes [37,41,72]. Nine esters were detected and quantified in HHP-treated blended juices and four in HHP–VFD-treated rehydrated juices, of which methyl and ethyl esters were dominant. HHP decreased (*p* < 0.05) the concentrations of esters in samples, which is consistent with the changes in most matrices, such as mango juice, red plum puree, and kiwifruit pulp beverage [6,54,73]. Methyl butyrate and ethyl butyrate contents decreased in response to pressure intensity. The precursors of esters in these matrices may be lipids and amino acids, and the key enzyme in the biosynthesis was the alcohol acyltransferase, the activity of which seemingly dropped under pressure [1,74,75]. Moreover, HHP–VFD further decreased (*p* < 0.05) the contents of esters, except for ethyl acetate, though VFD can extremely retain the ester content due to the lower temperature compared with other drying methods [76].

In addition, a two-way ANOVA was applied to four fresh, HHP3, VFD, and HHP3–VFD samples to analyze the effect of combined HHP and VFD treatments on the contents of volatile compounds (Table S1). The results of the analysis showed that, except for 10 volatile compounds, namely geranylacetone (*p* = 0.155), isovaleraldehyde (*p* = 0.165), valeraldehyde (*p* = 0.053), (*E*, *E*)-2,4-heptadienal (*p* = 0.821), (*E*)-2-nonenal (*p* = 0.515), 4-carene (*p* = 0.394), α, p-dimethylstyrene (*p* = 0.479), 1-pentanol (*p* = 0.223), (*E*)-3-Hexen-1-ol (*p* = 0.221), p-cymen-8-ol (*p* = 0.758), the interaction and main effect between HHP and VFD in terms of their effects on the contents of volatile compounds were significant (*p* < 0.05). These results further confirmed the interaction of HHP and VFD, rather than a one-way independent casual effect.

### 3.4. Effect of Different Analytical Methods on the Detection of Aroma-Active Compounds

Table S2 lists 27 odor-active compounds with DF ≥ 6 recognized by GC–O. These 27 compounds were categorized by odor characteristics into four groups according to the method of Castro et al. [77]. The first group consists of fragrant and sweet odorants (A5, A7, A10, B15, D3, E3, E4, E7, and F3 in Table 2 and Table S2) described as floral, nutty, and cheese. The second group mainly involves fruity odorants (A6, B3, B4, B5, B8, B11, B16, B17, B18, D4, and E8 in Table 2 and Table S2) expressed as citrus, orange peel, and green. The third group consists of woody and resinous odorants (B13, C1, and D5 in Table 2 and Table S2), with descriptors of waxy, pine, and oily. Compounds with pungent, sour, and sweaty (A3, E2, E5, and E6 in Table 2 and Table S2) constitute the last group described as off-flavor. 6-Methyl-5-hepten-2-one, isovaleraldehyde, hexanal, 1-nonanal, (*E*)-2-octenal, (*E*,*Z*)-2,6-nonadienal, (*E*)-3-hexen-1-ol, 1-octen-3-ol, propionic acid, butanoic acid, 2-methyl butanoic acid, hexanoic acid, and octanoic acid, were detected by assessors eight times at least in one sample, meaning that these 13 compounds were potentially critical contributors to the overall aroma of blended juices and rehydrated juices. Of them, 6-methyl-5-hepten-2-one, hexanal, 1-nonanal, (*E*,*Z*)-2,6-nonadienal, butanoic acid, and 2-methyl butanoic acid were reported as aroma-active compounds contributing to the aroma profile of mangoes of different varieties [58,78]. Hexanal and (*E*)-2-octanal were recognized as the most powerful odor-active compounds in jujube fruits from three cultivars, on the basis of both GC–O and OAV [79]. In another jujube cultivar named muzao, the aroma-impact compounds that affected the aroma profile were hexanoic acid and octanoic acid [80]. Furthermore, hexanal, (*E*)-3-hexen-1-ol, and 1-octen-3-ol were described as the most characteristic compounds of the variety of pumpkin [81]. However, DF analysis may not always directly relate to the aroma-active compounds, since it can only reveal the frequency, but lacks intensity. Therefore, the verification of aroma-active compounds shall also depend on OAV.

OAV is a reasonable tool for aroma potency assessment based on the equilibrium between air and the food matrix. Table S2 presents the 18 volatile compounds with OAV ≥ 1 and their reliable odor thresholds reported before, indicating they make powerful contributions to the overall aroma. Aldehydes occupy the majority of these compounds since many aldehydes possess a low odor threshold [50]. The C5 aldehydes, as isovaleraldehyde and valeraldehyde detected here, tend to contribute a chemical/malty/green odor in pumpkin and mango [40,78]. When chain length exceeds C6, such as hexanal and 1-nonanal, aldehydes detected here have dual properties with descriptors of fruity/floral or fatty notes, depending on the concentration, the substrate, and the person who senses them. Aldehydes containing an aromatic ring, such as benzaldehyde in this study, are major components of jujube, pumpkin and other foods that mainly emit sweet and nutty aromas, such as that of newly crushed almonds [37,40]. Unsaturated aldehydes with both (*E*) and (*Z*) isomer conformations (e.g., (*E*)-2-hexenal, (*E*)-2-nonenal, (*E*)-2-octenal, and (*E*,*Z*)-2,6-nonadienal herein) have higher odor thresholds and are usually compounds that affect characteristics. The shorter-chain analogs provide green aromas. It is worth mentioning that the odor thresholds of both saturated aliphatic and unsaturated aldehydes seemingly decrease gradually with the prolonging of chain length. Floral-like β-ionone in VFD-treated or HHP–VFD-treated rehydrated juices had the highest OAV (1333.23 and 1520.79, respectively), followed by cucumber-like (*E*,*Z*)-2,6-nonadienal in fresh juice (1076.69). Generally, HHP does not considerably alter the OAV of most volatiles [82], but (*E*,*Z*)-2,6-nonadienal, which had the largest OAV in fresh juice, was undetected in HHP1 and HHP2 juices. (*E*,*Z*)-2,6-Nonadienal is formed from linoleic acid, which is the substrate of LOX, and the activity of LOX may change under different pressures and in different matrices [83].

**Figure 4:**
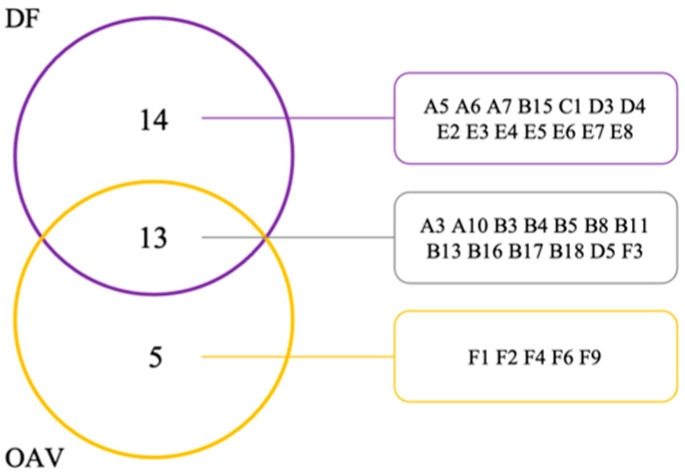
Distribution of aroma-active compounds identified in blended juices and rehydrated juices by DF and OAV (numbering refers to Table 2).

After comparison of the aroma-active compounds detected by DF and OAV (Figure 4), the joint analysis of each sample identified 4, 5, 4, 7, 9, and 8 aroma-active compounds from the fresh, HHP1, HHP2, HHP3, VFD, and HHP3–VFD samples, respectively. Hexanal, (*E*)-2-hexenal, and 1-octen-3-ol were found in all six samples, indicating they were potent and major aroma contributors and unaffected by processing. β-Ionone and β-cyclocitral were only detected in VFD-treated and HHP–VFD-treated samples and were screened as aroma-active compounds, and therefore, they can be used as indicators to identify whether a sample was treated with VFD or not. β-Ionone was derived from the degradation of β-carotenene under the enzymatic formation of carotenoid cleavage dioxygenase [84,85], and a higher level of β-carotenene degradation rate took place at a lower water activity [86]. As reported, β-ionone was positively associated with the sour flavor of freeze-dried tomatoes, while not associated with green or grassy flavor in fresh tomatoes [87,88]. Similarly, Xing et al. [89] compared the chemical compositions of fresh and dried purple perilla leaves and did not identify β-cyclocitral in fresh leaves, which appeared after drying due to the oxidation reaction, hydrolysis of glycosylated products, or the destruction of cell walls. Some high-OAV volatile compounds, such as ethyl butyrate and methyl benzoate, were not found by DF analysis. Among the components discriminated by the judges in GC–O test, 6-methyl-5-hepten-2-one, (*E*)-3-hexen-1-ol, propionic acid, butanoic acid, 2-methyl butanoic acid, hexanoic acid, and octanoic acid had OAVs < 1, suggesting they made limited contributions to the overall aroma. The differences between the two assessments were mainly due to the different principles applied [24]. OAV was calculated based on the odor threshold in water rather than the food matrix. In the actual food matrix, the presence of abundant chemical compositions significantly impacts the perception of aroma. Specifically, the aroma release will be intensified or restricted more or less by the interaction between food components and volatile compounds [90,91]. Thus, the combination of DF analysis and OAV is necessary to accurately identify the aroma-active compounds.

### 3.5. Flavor Profile

**Figure 5:**
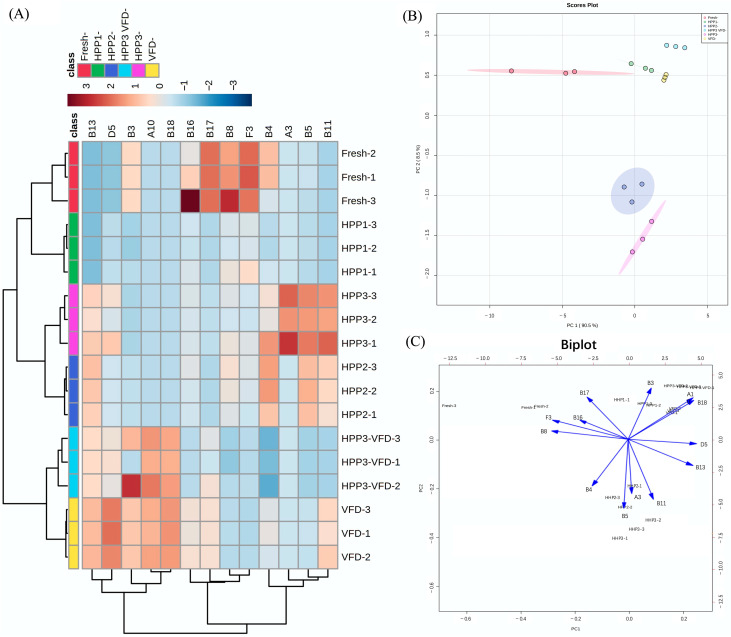
(**A**) Heat map and hierarchical clustering of 13 major aroma-active compounds in different processed blended juices and rehydrated juices. (**B**) Score plots and (**C**) biplots of PCA on the expressions of the major aroma-active compounds.

Heat map, hierarchical clustering, and PCA of the concentrations of the 13 major aroma-active compounds were performed to characterize the aroma profiles of blended juices with different treatments (Figure 5). Heat map and hierarchical clustering analysis (Figure 5A) demonstrate the profiles of 13 major aroma-active compounds present in blended juices with different processing methods. The color intensity (blue or red) is consistent with the concentrations of aroma-active compounds in Table 2. It indicates that HHP1 is closer to fresh blended juice in terms of the association in cluster analysis. Additionally, the VFD- and non-VFD-treated blended juices were divided into two categories. Qualitative and quantitative data of the six samples were subjected to PCA to compare differences between the fresh and treated blended juices. The principal component score plot and biplot in Figure 5B,C show that the two PCs can explain 99% of the variability. Fresh blended juice (red circle in Figure 5B) was clearly distinguished from all the others, suggesting the typical aroma was altered during processing. HHP1 juice (green circle in Figure 5B) was much closer to VFD- and HHP–VFD-treated rehydrated juices (yellow and blue circles in Figure 5B, respectively) compared with HHP2 and HHP3 juices. Euclidean distance in score plots (Figure 5B) agreed well with the hierarchical clustering results (Figure 5A). β-Ionone and β-cyclocitral (B18 and A10 in Figure 5C) were only directed to VFD- and HHP–VFD-treated rehydrated juices instead of blended juices, indicating they can be used as indicators to distinguish samples treated with VFD or not. However, the concrete aroma description of either PC, which may alter the specific sensory aroma attributes, calls for further research and discussion.

### 3.6. Comparative Sensory Analysis of Blended Juices with Different Processing Methods

**Figure 6:**
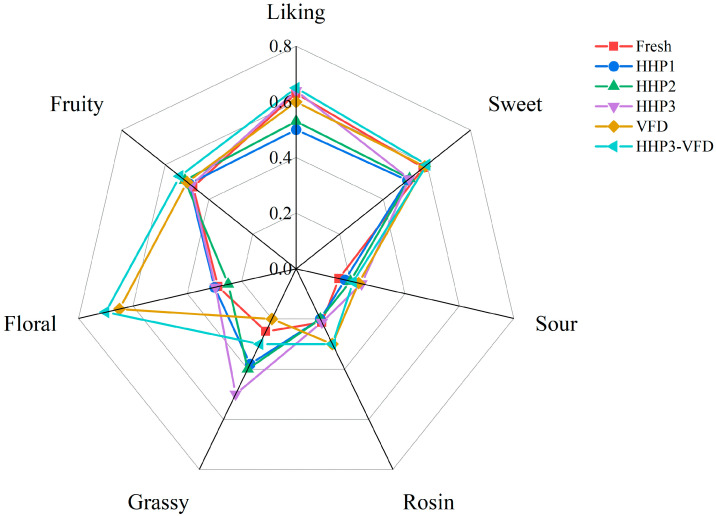
Sensory characteristics of blended juices prepared by different processing methods via QDA.

Quantitative descriptive analysis (QDA) including six aroma notes and overall liking was used to distinguish the sensory quality with different processing methods (Figure 6). Six aroma notes (sweet, sour, rosin, grassy, floral, and fruity) were put forward in pre-evaluation to quantify sensory differences. No significant differences were found in the scores of sour or fruity among the six samples (*p* < 0.05). Noticeably, HHP-treated blended juices showed a stronger grassy aroma, the intensity of which was strengthened as pressure increased. Reportedly, HHP-treated kiwifruit pulp can cause more intense grassy and fresh notes, which may be contributed by hexanal [54]. HHP–VFD-treated rehydrated juices showed higher scores in rosin and floral, which conformed to the concentrations of 3-carene and β-ionone during VFD, respectively. β-Ionone was also described as a floral note and judged to be stronger in freeze-dried tomatoes [92]. As for the sweet aroma note and overall liking, HHP–VFD-treated rehydrated juices relative to the HHP-treated samples were graded with a very close score to the fresh juices. These findings are in accord with the results of hierarchical clustering and PCA. A study in candied green plum also showed that the combination of HHP and osmotic drying retained the flavor of fresh samples [93]. Thus, the 13 major aroma-active compounds can be predicted to characterize the flavor of blended juices.

## 4. Conclusions

Compared to HHP or VFD alone, the combination of HHP–VFD resulted in a better retention of flavor quality in blended juices. HHP treatment at 200, 400, and 600 MPa did not lead to significant changes in the color of blended juices but decreases in the total volatiles and changes in sensory quality. The HHP–VFD treatment changed the color, microstructure and caused a decrease in total volatiles. Moreover, HHP–VFD resulted in the highest terpene and ketone contents of blended juices among the different treatments, especially the aroma-active compounds β-ionone and β-cyclocitral, so they can be recognized as discriminant indicators of HHP–VFD treatment. Rehydrated juice from HHP–VFD-treated rehydrated juices can better retain the flavor quality of fresh juice, indicating a possible application for the non-thermal processing combination of HHP and VFD in the fruit and vegetable juice industry. Although we maximized flavor retention based on the extended shelf life, further research in this field is required to apply this non-thermal processing combination into food and other matrices.

## Figures and Tables

**Table 1:** Color parameters in blended juice and cubes treated by HHP and VFD.

		L*	a*	b*	C*	h^0^	ΔE
Juices	0 MPa (Fresh)	36.58 ± 0.25 c	6.05 ± 0.03b c	17.45 ± 0.29 bc	18.47 ± 0.27 bc	70.88 ± 0.35 bc	-
	200 MPa (HHP1)	36.81 ± 0.03 c	6.20 ± 0.26 b	17.26 ± 0.13 c	18.34 ± 0.21 c	70.24 ± 0.63 c	0.46 ± 0.12 c
	400 MPa (HHP2)	36.86 ± 0.14 c	6.15 ± 0.02b c	17.69 ± 0.43 b	18.73 ± 0.40 bc	70.81 ± 0.48 bc	0.45 ± 0.43 c
	600 MPa (HHP3)	36.70 ± 0.06 c	5.96 ± 0.09 c	17.81 ± 0.01 b	18.78 ± 0.03 b	71.50 ± 0.26 b	0.48 ± 0.26 c
	VFD-treated rehydrated juices	40.98 ± 0.02 b	6.68 ± 0.11 a	21.52 ± 0.05 a	22.54 ± 0.04 a	72.77 ± 0.29 a	5.62 ± 0.03 b
	HHP3–VFD-treated rehydrated juices	41.25 ± 0.04 a	6.85 ± 0.07 a	21.78 ± 0.10 a	22.83 ± 0.11 a	72.53 ± 0.12 a	6.01 ± 0.09 a
Cubes	0 MPa (VFD)	62.36 ± 0.17	17.52 ± 0.08	52.82 ± 0.14	55.65 ± 0.16	71.65 ± 0.04	-
	600 MPa (HHP3–VFD)	63.77 ± 0.68	17.94 ± 0.37	53.00 ± 0.33	55.95 ± 0.43	71.30 ± 0.25	1.49 ± 0.62

Different minuscule letters in the same column mean significant difference at *p* < 0.05. “-“ means the sample used as a reference in the calculation of the ΔE value. HHP1, blended juice treated with HHP at 200 MPa; HHP2, blended juice treated with HHP at 400 MPa; HHP3, blended juice treated with HHP at 600 MPa; VFD, blended cube of fresh juice treated after VFD; HHP3–VFD, blended cube of HHP3 juice treated after VFD (the same below).

**Table 2:** Volatile compounds in blended juices treated by HHP and VFD recognized by HS-SPME-GC–MS.

No.	Compounds	CAS	LRI ^1^	Concentration (μg/kg) ^2^						Identification ^3^	Origin ^4^
				HHP-Treated				HHP–VFD-Treated			
				0 MPa (Fresh)	200 MPa (HHP1)	400 MPa (HHP2)	600 MPa (HHP3)	0 MPa (VFD)	600 MPa (HHP3–VFD)		
	Ketones										
A1	Acetone	67-64-1	755	4.09 ± 0.73 a	2.40 ± 0.05 b	1.97 ± 0.11 c	1.89 ± 0.23 c	1.23 ± 0.12 d	n.d.	MS, LRI	Mango
A2	2-Butanone	78-93-3	888	n.d.	2.06 ± 0.30 a	n.d.	n.d.	n.d.	n.d.	MS, LRI	Pumpkin
A3	Ethyl vinyl ketone	1629-58-9	1010	n.d.	n.d.	n.d.	2.26 ± 0.63 a	n.d.	n.d.	MS, LRI	Jujube, mango
A4	1-(1,3-Dimethyl-3-cyclohexen-1-yl) ethanone	51733-68-7	1137	4.33 ± 0.15 a	3.44 ± 0.32 b	2.54 ± 0.13 c	1.79 ± 0.32 d	n.d.	n.d.	MS	Other
A5	Acetoin	513-86-0	1274	5.57 ± 0.96 b	1.91 ± 0.67 c	1.86 ± 0.23 c	2.23 ± 0.34 c	n.d.	18.60 ± 2.03 a	MS, LRI	Pumpkin
A6	6-Methyl-5-hepten-2-one	110-93-0	1325	1.01 ± 0.08 d	1.13 ± 0.21 c	2.45 ± 0.10 b	1.23 ± 0.11 c	4.15 ± 0.14 a	2.52 ± 0.03 b	MS, LRI	Jujube, pumpkin, mango
A7	4’-Methyl acetophenone	122-00-9	1751	n.d.	n.d.	n.d.	1.28 ± 0.08 a	n.d.	n.d.	MS, LRI	Pumpkin
A8	β-Damascenone	23726-93-4	1771	n.d.	n.d.	1.50 ± 0.33 c	1.62 ± 0.11 b	n.d.	3.14 ± 0.42 a	MS, LRI	Pumpkin
A9	Geranylacetone	3796-70-1	1822	n.d.	n.d.	n.d.	n.d.	4.26 ± 0.44 b	6.18 ± 1.66 a	MS, LRI	Pumpkin, mango
A10	β-Ionone	79-77-6	1896	n.d.	n.d.	n.d.	n.d.	9.33 ± 0.73 b	10.65 ± 1.91 a	MS, LRI	Jujube, pumpkin
	Subtotal			15.00 ± 1.92 c	10.94 ± 1.55 d	10.32 ± 0.90 d	12.30 ± 1.82 d	18.97 ± 1.43 b	41.09 ± 6.05 a		
	**Aldehydes**										
B1	Acetaldehyde	75-07-0	682	7.11 ± 1.04 a	2.17 ± 0.80 b	2.35 ± 0.40 b	2.19 ± 0.67 b	1.77 ± 0.26 b	n.d.	MS, LRI	Mango
B2	2-Methylbutyraldehyde	96-17-3	866	n.d.	n.d.	n.d.	n.d.	2.42 ± 1.83 a	n.d.	MS, LRI	Pumpkin
B3	Isovaleraldehyde	590-86-3	878	4.10 ± 0.39 ab	1.69 ± 0.38 b	2.22 ± 0.35 b	1.76 ± 0.26 b	4.71 ± 0.71 a	5.18 ± 4.51 a	MS, LRI	Mango
B4	Valeraldehyde	110-62-3	928	15.59 ± 3.80 ab	9.35 ± 1.06 c	19.41 ± 1.95 a	15.85 ± 4.46 ab	11.84 ± 1.17 bc	4.29 ± 1.06 d	MS, LRI	Pumpkin
B5	Hexanal	66-25-1	1070	22.26 ± 2.43 c	17.74 ± 1.38 c	77.88 ± 4.70 b	102.59 ± 7.16 a	23.12 ± 1.69 c	8.81 ± 0.90 d	MS, LRI	Jujube, pumpkin, mango
B6	3-Methyl-2-butenal	107-86-8	1199	n.d.	n.d.	n.d.	n.d.	4.78 ± 0.63 a	3.56 ± 0.30 b	MS, LRI	Pumpkin
B7	Heptaldehyde	111-71-7	1174	n.d.	n.d.	n.d.	2.96 ± 0.23 a	n.d.	1.76 ± 0.14 b	MS, LRI	Pumpkin
B8	(*E*)-2-Hexenal	6728-26-3	1207	393.53 ± 96.22 a	146.01 ± 27.53 b	145.29 ± 24.56 b	148.52 ± 26.43 b	86.48 ± 4.38 c	56.47 ± 35.73 d	MS, LRI	Jujube, pumpkin, mango
B9	Furfural	98-01-1	1439	8.24 ± 1.19 a	2.75 ± 0.78 b	3.27 ± 0.61 b	2.77 ± 0.59 b	2.72 ± 0.59 b	3.12 ± 0.15 b	MS, LRI	Jujube, pumpkin
B10	(*E*)-2-Heptenal	18829-55-5	1309	5.24 ± 0.61 c	1.36 ± 0.06 d	10.24 ± 0.54 ab	8.61 ± 0.93 b	11.32 ± 0.07 a	6.00 ± 3.48 c	MS, LRI	Jujube, mango
B11	1-Nonanal	124-19-6	1377	n.d.	n.d.	1.89 ± 0.28 b	3.30 ± 0.48 a	1.96 ± 0.30 b	n.d.	MS, LRI	Jujube, pumpkin, mango
B12	5-Ethyl-1-cyclopentene-1-carboxaldehyde	36431-60-4	1393	n.d.	n.d.	3.29 ± 0.13 b	3.52 ± 0.27 a	n.d.	n.d.	MS	Other
B13	(*E*)-2-Octenal	2548-87-0	1408	n.d.	n.d.	8.18 ± 0.38 b	7.49 ± 0.65 c	8.95 ± 0.19 a	7.08 ± 0.92 c	MS, LRI	Jujube
B14	(*E*, *E*)-2,4-Heptadienal	4313-03-5	1446	n.d.	n.d.	n.d.	n.d.	4.07 ± 0.60 a	4.01 ± 0.62 a	MS, LRI	Jujube, pumpkin
B15	Benzaldehyde	100-52-7	1499	58.31 ± 5.12 a	29.67 ± 2.51 c	39.17 ± 6.10 b	32.94 ± 3.97 bc	12.24 ± 0.08 d	11.80 ± 2.68 d	MS, LRI	Jujube, pumpkin
B16	(*E*)-2-Nonenal	18829-56-6	1511	17.67 ± 16.53 a	2.70 ± 1.08 b	3.73 ± 1.15 b	3.80 ± 0.39 b	7.37 ± 1.06 ab	n.d.	MS, LRI	Jujube, mango
B17	(*E*,*Z*)-2,6-Nonadienal	557-48-2	1561	21.53 ± 3.78 a	n.d.	n.d.	1.84 ± 0.16 d	8.43 ± 0.12 b	5.58 ± 3.43 c	MS, LRI	Mango
B18	β-Cyclocitral	432-25-7	1589	n.d.	n.d.	n.d.	n.d.	7.80 ± 0.23 a	7.17 ± 0.57 b	MS, LRI	Pumpkin
B19	5-Hydroxymethylfurfural	67-47-0	2499	n.d.	n.d.	n.d.	n.d.	1.69 ± 0.30 a	n.d.	MS, LRI	Mango
	Subtotal			553.58 ± 131.11 a	213.44 ± 35.58 c	316.92 ± 41.15 b	338.14 ± 46.65 b	201.67 ± 14.21 c	124.83 ± 54.46 d		
	**Terpenes**										
C1	3-Carene	13466-78-9	1126	10.23 ± 2.44 c	9.26 ± 2.31 c	10.10 ± 1.06 c	8.64 ± 1.87 c	101.94 ± 1.09 b	162.76 ± 19.51 a	MS, LRI	Mango
C2	α-Phellandrene	99-83-2	1133	n.d.	n.d.	n.d.	n.d.	2.18 ± 0.12 b	3.79 ± 0.07 a	MS, LRI	Jujube, mango
C3	Myrcene	123-35-3	1150	n.d.	n.d.	n.d.	n.d.	2.48 ± 0.16 b	4.01 ± 1.28 a	MS, LRI	Jujube, mango
C4	β-Thujene	28634-89-1	1146	n.d.	n.d.	n.d.	n.d.	2.40 ± 0.19 b	3.97 ± 0.40 a	MS, LRI	Mango
C5	4-Carene	29050-33-7	1166	15.24 ± 4.86 ab	11.15 ± 1.94 bc	6.95 ± 1.83 cd	2.43 ± 0.26 d	19.18 ± 4.51 a	3.35 ± 0.55 d	MS, LRI	Mango
C6	DL-Limonene	138-86-3	1175	n.d.	n.d.	n.d.	n.d.	6.55 ± 1.23 b	14.95 ± 2.93 a	MS, LRI	Jujube, mango
C7	Terpinolene	586-62-9	1266	15.28 ± 3.78 b	11.11 ± 0.65 c	n.d.	n.d.	n.d.	92.78 ± 5.20 a	MS, LRI	Jujube, mango
C8	α, p-Dimethylstyrene	1195-32-0	1416	n.d.	n.d.	n.d.	n.d.	6.95 ± 0.28 a	8.51 ± 5.15 a	MS, LRI	Mango
	Subtotal			40.75 ± 11.08 c	31.52 ± 4.90 c	17.05 ± 2.89 d	11.07 ± 2.13 d	141.68 ± 7.58 b	294.12 ± 35.08 a		
	**Alcohols**										
D1	1-Penten-3-ol	616-25-1	1158	n.d.	n.d.	n.d.	n.d.	2.33 ± 0.10 a	n.d.	MS, LRI	Jujube, pumpkin
D2	2-Methyl-1-butanol	137-32-6	1202	n.d.	n.d.	n.d.	n.d.	n.d.	10.81 ± 0.98 a	MS, LRI	Jujube, mango
D3	1-Pentanol	71-41-0	1244	6.18 ± 0.19 ab	2.50 ± 0.91 c	5.64 ± 0.64 b	8.10 ± 2.50 a	n.d.	n.d.	MS, LRI	Jujube, pumpkin, mango
D4	(*E*)-3-Hexen-1-ol	928-97-2	1370	13.54 ± 0.87 a	5.92 ± 1.49 c	6.43 ± 0.84 c	9.76 ± 2.12 b	2.02 ± 0.08 d	n.d.	MS, LRI	Jujube, mango
D5	1-Octen-3-ol	3391-86-4	1435	5.15 ± 1.81 d	7.43 ± 0.32 c	8.33 ± 2.38 c	10.89 ± 1.95 b	17.07 ± 0.75 a	10.43 ± 0.92 b	MS, LRI	Jujube, pumpkin, mango
D6	p-Cymen-8-ol	1197-01-9	1798	n.d.	n.d.	n.d.	n.d.	9.79 ± 0.97 a	10.08 ± 1.98 a	MS, LRI	Mango
D7	Benzyl alcohol	100-51-6	1820	n.d.	n.d.	n.d.	n.d.	1.26 ± 0.22 b	2.37 ± 1.01 a	MS, LRI	Jujube
D8	Carveol	99-48-9	1857	n.d.	n.d.	n.d.	n.d.	43.31 ± 1.05 a	26.11 ± 6.56 b	MS, LRI	Mango
	Subtotal			24.87 ± 2.87 d	15.85 ± 2.72 e	20.40 ± 3.86 e	28.75 ± 6.57 c	75.78 ± 3.17 a	59.80 ± 11.45 b		
	**Acids**										
E1	Acetic acid	64-19-7	1435	191.64 ± 32.42 a	59.12 ± 25.37 b	64.19 ± 4.98 b	63.49 ± 16.09 b	4.84 ± 0.90 c	8.38 ± 2.40 c	MS, LRI	Jujube, pumpkin
E2	Propionic acid	79-09-4	1522	5.02 ± 0.63 a	2.15 ± 0.16 b	2.05 ± 0.38 b	1.69 ± 0.28 b	n.d.	n.d.	MS, LRI	Jujube
E3	Butanoic acid	107-92-6	1608	9.44 ± 2.00 a	2.49 ± 0.79 bc	2.90 ± 0.70 b	2.46 ± 0.64 bc	1.03 ± 0.15 c	3.24 ± 0.77 b	MS, LRI	Pumpkin, mango
E4	2-Methyl butanoic acid	116-53-0	1647	17.30 ± 3.74 a	5.63 ± 1.85 b	6.88 ± 1.41 b	5.58 ± 1.19 b	n.d.	n.d.	MS, LRI	Pumpkin, mango
E5	Valeric acid	109-52-4	1707	12.05 ± 1.47 a	3.67 ± 0.94 b	4.96 ± 1.18 b	4.23 ± 0.77 b	n.d.	n.d.	MS, LRI	Pumpkin
E6	Hexanoic acid	142-62-1	1797	115.90 ± 17.58 a	40.13 ± 10.51 b	60.32 ± 15.41 b	47.12 ± 8.68 b	8.57 ± 1.36 c	12.75 ± 2.81 c	MS, LRI	Jujube, pumpkin, mango
E7	Heptanoic acid	111-14-8	1884	37.33 ± 3.83 a	13.58 ± 3.51 b	17.89 ± 4.80 b	14.81 ± 2.06 b	6.21 ± 1.44 c	6.39 ± 2.41 c	MS, LRI	Jujube
E8	Octanoic acid	124-07-2	2009	19.34 ± 0.92 a	7.81 ± 1.78 bc	9.69 ± 2.60 b	8.44 ± 1.25 bc	7.05 ± 2.19 bc	5.81 ± 2.72 c	MS, LRI	Jujube, pumpkin, mango
E9	Benzoic acid	65-85-0	2391	16.57 ± 3.67 a	4.30 ± 1.01 b	4.96 ± 2.04 b	5.33 ± 0.88 b	n.d.	n.d.	MS, LRI	Jujube, pumpkin
	Subtotal			424.59 ± 66.26 a	138.88 ± 45.92 b	173.84 ± 33.50 b	153.15 ± 31.84 b	27.70 ± 6.04 c	36.57 ± 11.11 c		
	**Esters**										
F1	Methyl acetate	79-20-9	764	4.85 ± 1.18 a	1.82 ± 0.47 b	1.81 ± 0.22 b	2.01 ± 0.21 b	1.23 ± 0.01 b	n.d.	MS, LRI	Jujube, pumpkin
F2	Ethyl acetate	141-78-6	852	40.19 ± 14.98 a	10.63 ± 8.20 b	5.75 ± 1.10 b	5.99 ± 3.12 b	5.20 ± 1.67 b	10.4 ± 5.68 b	MS, LRI	Jujube
F3	Methyl butyrate	623-42-7	986	72.25 ± 4.34 a	19.45 ± 9.50 b	12.61 ± 2.79 b	11.73 ± 4.01 b	1.62 ± 0.10 c	2.82 ± 1.51 c	MS, LRI	Jujube, mango
F4	Ethyl butyrate	105-54-4	1028	97.67 ± 49.16 a	14.30 ± 13.10 b	5.49 ± 2.03 b	3.94 ± 1.86 b	n.d.	n.d.	MS, LRI	Jujube, mango
F5	Methyl valerate	624-24-8	1076	9.54 ± 1.37 a	4.08 ± 0.71 c	5.77 ± 0.86 b	5.25 ± 0.81 bc	n.d.	n.d.	MS, LRI	Jujube
F6	Isoamyl acetate	123-92-2	1113	4.72 ± 0.75 a	n.d.	n.d.	n.d.	n.d.	n.d.	MS, LRI	Mango
F7	Methyl hexanoate	106-70-7	1178	30.90 ± 3.93 a	15.6 ± 1.94 c	23.71 ± 3.11 b	18.08 ± 2.41 c	3.88 ± 0.30 d	n.d.	MS, LRI	Jujube, mango
F8	Methyl heptanoate	106-73-0	1278	3.79 ± 0.19 a	2.25 ± 0.15 c	3.43 ± 0.41 a	n.d.	2.72 ± 0.46 b	2.44 ± 0.17 bc	MS, LRI	Jujube
F9	Methyl benzoate	93-58-3	1595	9.77 ± 1.70 a	5.39 ± 0.40 c	7.71 ± 1.64 b	5.98 ± 0.57 bc	6.27 ± 0.04 bc	5.25 ± 0.95 c	MS, LRI	Jujube
F10	3-Phenylpropionic acid methyl ester	103-25-3	1854	n.d.	1.64 ± 0.13 b	2.39 ± 0.49 a	1.85 ± 0.25 b	n.d.	n.d.	MS, LRI	Jujube
	Subtotal			273.68 ± 77.60 a	75.16 ± 34.60 b	68.67 ± 12.65 b	54.83 ± 13.24 c	20.92 ± 2.58 d	20.91 ± 8.31 d		
	Total			1332.47 ± 290.84 a	485.79 ± 125.27 c	607.2 ± 94.95 b	598.24 ± 102.25 b	486.72 ± 35.01 c	577.32 ± 126.46 b		

^1^ LRI: linear retention index on DB-WAX column. ^2^ n.d. means not detected since the concentration of the given compound was below the detection limit. ^3^ Identification, volatiles were identified as follows: LRI, comparing LRI calculated herein with that in open access data of the NIST WebBook; MS, mass spectrum comparisons with NIST10 database. ^4^ Origin, sources of volatile compounds in the literature. Different minuscule letters in the same row mean significant difference at *p* < 0.05.

## Data Availability

The datasets generated for this study are available on request to the corresponding author.

## Supplementary Materials

The following are available online at https://www.mdpi.com/article/10.3390/foods10123151/s1, Table S1: The result of testing HHP*VFD between-subject effects in fresh, HHP3, VFD, and HHP3–VFD samples, Table S2: Identification of aroma-active compounds in blended juices treated by HHP and VFD by DF and OAV analyses.

## References

[1] Lomeli-Martin A., Maria Martinez L., Welti-Chanes J., Escobedo-Avellaneda Z. (2021). Induced changes in aroma compounds of foods treated with high hydrostatic pressure: A review. Foods.

[2] De Marchi F., Aprea E., Endrizzi I., Charles M., Betta E., Corollaro M.L., Cappelletti M., Ferrentino G., Spilimbergo S., Gasperi F. (2015). Effects of pasteurization on volatile compounds and sensory properties of coconut (*Cocos nucifera* L.) water: Thermal vs. high-pressure carbon dioxide pasteurization. Food Bioprocess Technol..

[3] Luo D., Pang X., Xu X., Bi S., Zhang W., Wu J. (2018). Identification of cooked off-flavor components and analysis of their formation mechanisms in melon juice during thermal processing. J. Agric. Food Chem..

[4] Podolak R., Whitman D., Black D.G. (2020). Factors affecting microbial inactivation during high pressure processing in juices and beverages: A review. J. Food Prot..

[5] Oey I., Lille M., van Loey A., Hendrickx M. (2008). Effect of high-pressure processing on colour, texture and flavour of fruit- and vegetable-based food products: A review. Trends Food Sci. Technol..

[6] Zhang W., Dong P., Lao F., Liu J., Liao X., Wu J. (2019). Characterization of the major aroma-active compounds in Keitt mango juice: Comparison among fresh, pasteurization and high hydrostatic pressure processing juices. Food Chem..

[7] Yi J., Kebede B.T., Doan Ngoc Hai D., Buve C., Grauwet T., Van Loey A., Hu X., Hendrickx M. (2017). Quality change during high pressure processing and thermal processing of cloudy apple juice. LWT Food Sci. Technol..

[8] Liu F., Zhang X., Zhao L., Wang Y., Liao X. (2016). Potential of high-pressure processing and high-temperature/short-time thermal processing on microbial, physicochemical and sensory assurance of clear cucumber juice. Innov. Food Sci. Emerg. Technol..

[9] Baxter I.A., Easton K., Schneebeli K., Whitfield F.B. (2005). High pressure processing of Australian navel orange juices: Sensory analysis and volatile flavor profiling. Innov. Food Sci. Emerg. Technol..

[10] Gomes W.F., Tiwari B.K., Rodriguez O., de Brito E.S., Narciso Fernandes F.A., Rodrigues S. (2017). Effect of ultrasound followed by high pressure processing on prebiotic cranberry juice. Food Chem..

[11] Zhao L., Qin X., Han W., Wu X., Wang Y., Hu X., Ling J., Liao X. (2018). Novel application of CO_2_-assisted high pressure processing in cucumber juice and apple juice. LWT Food Sci. Technol..

[12] Zhao L., Wang Y., Hu X., Sun Z., Liao X. (2016). Korla pear juice treated by ultrafiltration followed by high pressure processing or high temperature short time. LWT Food Sci. Technol..

[13] Shishehgarha F., Makhlouf J., Ratti C. (2002). Freeze-drying characteristics of strawberries. Dry. Technol..

[14] Ul Hasan M., Malik A.U., Ali S., Imtiaz A., Munir A., Amjad W., Anwar R. (2019). Modern drying techniques in fruits and vegetables to overcome postharvest losses: A review. J. Food Process. Preserv..

[15] Ansar, Nazaruddin, Azis A.D. (2020). New frozen product development from strawberries (*Fragaria Ananassa* Duch.). Heliyon.

[16] Wojdylo A., Lech K., Nowicka P., Hernandez F., Figiel A., Antonio Carbonell-Barrachina A. (2019). Influence of different drying techniques on phenolic compounds, antioxidant capacity and colour of *Ziziphus jujube* Mill. fruits. Molecules.

[17] Perfilova O.V., Akishin D.V., Vinnitskaya V.F., Danilin S.I., Olikainen O.V. (2020). Use of vegetable and fruit powder in the production technology of functional food snacks. IOP Conf. Ser. Earth Environ. Sci..

[18] Gonzalez-Pena D., Colina-Coca C., Char C.D., Pilar Cano M., de Ancos B., Sanchez-Moreno C. (2013). Hyaluronidase inhibiting activity and radical scavenging potential of flavonols in processed onion. J. Agric. Food Chem..

[19] Colina-Coca C., de Ancos B., Sanchez-Moreno C. (2014). Nutritional composition of processed onion: *S*-alk(en)yl-L-cysteine sulfoxides, organic acids, sugars, minerals, and vitamin C. Food Bioprocess Technol..

[20] Xu X., Bao Y., Wu B., Lao F., Hu X., Wu J. (2019). Chemical analysis and flavor properties of blended orange, carrot, apple and Chinese jujube juice fermented by selenium-enriched probiotics. Food Chem..

[21] Laurentin A., Edwards C.A. (2003). A microtiter modification of the anthrone-sulfuric acid colorimetric assay for glucose-based carbohydrates. Anal. Biochem..

[22] Zhang W., Liang L., Pan X., Lao F., Liao X., Wu J. (2021). Alterations of phenolic compounds in red raspberry juice induced by high-hydrostatic-pressure and high-temperature short-time processing. Innov. Food Sci. Emerg. Technol..

[23] Zhang L., Qiao Y., Wang C., Liao L., Shi D., An K., Hu J., Wang J., Shi L. (2020). Influence of high hydrostatic pressure pretreatment on properties of vacuum-freeze dried strawberry slices. Food Chem..

[24] Pang X., Guo X., Qin Z., Yao Y., Hu X., Wu J. (2012). Identification of aroma-active compounds in jiashi muskmelon juice by GC-O-MS and OAV calculation. J. Agric. Food Chem..

[25] Pan X., Zhang W.T., Lao F., Mi R.F., Liao X.J., Luo D.S., Wu J.H. (2021). Isolation and identification of putative precursors of the volatile sulfur compounds and their inhibition methods in heat-sterilized melon juices. Food Chem..

[26] Bi S., Xu X.X., Luo D.S., Lao F., Pang X.L., Shen Q., Hu X.S., Wu J.H. (2020). Characterization of key aroma compounds in raw and roasted peas (*Pisum sativum* L.) by application of instrumental and sensory techniques. J. Agric. Food Chem..

[27] Guth H. (1997). Quantitation and sensory studies of character impact odorants of different white wine varieties. J. Agric. Food Chem..

[28] Stone H., Sidel J., Oliver S., Woosley A., Singleton R.C. (1974). Sensory evaluation by quantitative description analysis. Food Technol..

[29] Stone H., Sidel J.L., Taylor S. (1993). Sensory evaluation practices. Food Science and Technology.

[30] Koutchma T., Popovic V., Ros-Polski V., Popielarz A. (2016). Effects of ultraviolet light and high-pressure processing on quality and health-related constituents of fresh juice products. Compr. Rev. Food Sci. Food Saf..

[31] Izli N., Izli G., Taskin O. (2017). Influence of different drying techniques on drying parameters of mango. Food Sci. Technol..

[32] Stamenkovic Z., Pavkov I., Radojcin M., Horecki A.T., Keselj K., Kovacevic D.B., Putnik P. (2019). Convective drying of fresh and frozen raspberries and change of their physical and nutritive properties. Foods.

[33] Izli N., Polat A. (2019). Freeze and convective drying of quince (*Cydonia oblonga* Miller.): Effects on drying kinetics and quality attributes. Heat Mass Transfer.

[34] Zvitov-Ya’ari R., Nussinovitch A. (2014). Browning prevention in rehydrated freeze-dried non-blanched potato slices by electrical treatment. LWT Food Sci. Technol..

[35] Dash K.K., Balasubramaniam V.M., Kamat S. (2019). High pressure assisted osmotic dehydrated ginger slices. J. Food Eng..

[36] Swami Hulle N.R., Rao P.S. (2016). Effect of High pressure pretreatments on structural and dehydration characteristics of aloe vera (*Aloe barbadensis* Miller) cubes. Dry. Technol..

[37] Pu Y., Ding T., Lv R., Cheng H., Liu D. (2018). Effect of drying and storage on the volatile compounds of jujube fruit detected by electronic nose and GC-MS. Food Sci. Technol. Res..

[38] Reche J., Hernandez F., Almansa M.S., Carbonell-Barrachina A.A., Legua P., Amoros A. (2019). Effects of organic and conventional farming on the physicochemical and functional properties of jujube fruit. LWT Food Sci. Technol..

[39] Kreck M., Patz C.D., Ludwig M., Degenhardt A., Paschold P., Dietrich H. (2004). Influence of variety on carotinoid content and flavour in pumpkin juice. Dtsch. Lebensm.-Rundsch..

[40] Li Y. (2010). Solid phase microextraction followed by GC-MS analysis of volatile flavor compounds in fresh pumpkin and pumpkin juice. Food Sci..

[41] Pino J.A., Mesa J., Munoz Y., Marti M.P., Marbot R. (2005). Volatile components from mango (*Mangifera indica* L.) cultivars. J. Agric. Food Chem..

[42] Bhardwaj R.L., Pandey S. (2011). Juice blends-a way of utilization of under-utilized fruits, vegetables, and spices: A review. Crit. Rev. Food Sci. Nutr..

[43] Hjelmeland A.K., King E.S., Ebeler S.E., Heymann H. (2013). Characterizing the chemical and sensory profiles of United States Cabernet Sauvignon wines and blends. Am. J. Enol. Vitic..

[44] Bi S., Sun S., Lao F., Liao X., Wu J. (2020). Gas chromatography-mass spectrometry combined with multivariate data analysis as a tool for differentiating between processed orange juice samples on the basis of their volatile markers. Food Chem..

[45] Chen X., Qin W., Ma L., Xu F., Jin P., Zheng Y. (2015). Effect of high pressure processing and thermal treatment on physicochemical parameters, antioxidant activity and volatile compounds of green asparagus juice. LWT Food Sci. Technol..

[46] Santos M.C., Nunes C., Rocha M.A.M., Rodrigues A., Rocha S.M., Saraiva J.A., Coimbra M.A. (2015). High pressure treatments accelerate changes in volatile composition of sulphur dioxide-free wine during bottle storage. Food Chem..

[47] Krokida M.K., Philippopoulos C. (2006). Volatility of apples during air and freeze drying. J. Food Eng..

[48] Chin S.T., Nazimah S.A.H., Quek S.Y., Man Y.B.C., Rahman R.A., Hashim D.M. (2008). Changes of volatiles’ attribute in durian pulp during freeze- and spray-drying process. LWT Food Sci. Technol..

[49] Dong W., Hu R., Chu Z., Zhao J., Tan L. (2017). Effect of different drying techniques on bioactive components, fatty acid composition, and volatile profile of robusta coffee beans. Food Chem..

[50] Parker J.K. (2015). Introduction to aroma compounds in foods. Flavour Development, Analysis and Perception in Food and Beverages.

[51] Hnin K.K., Zhang M., Wang B. (2021). Impact of different FD-related drying methods on selected quality attributes and volatile compounds of rose flavored yogurt melts. Dry. Technol..

[52] Musso L., Scaglia B., Al Haj G., Arnold N.A., Adani F., Scari G., Dallavalle S., Iriti M. (2017). Chemical characterization and nematicidal activity of the essential oil of *Nepeta nuda* L. ssp *pubescens* and *Nepeta curviflora* Boiss. from lebanon. J. Essent. Oil Bear. Plants.

[53] Belitz H.-D., Grosch W., Schieberle P. (2009). Lipids. Food Chemistry.

[54] Chen Y., Feng X., Ren H., Yang H., Liu Y., Gao Z., Long F. (2020). Changes in physicochemical properties and volatiles of kiwifruit pulp beverage treated with high hydrostatic pressure. Foods.

[55] Anthon G.E., Barrett D.M. (2003). Thermal inactivation of lipoxygenase and hydroperoxytrienoic acid lyase in tomatoes. Food Chem..

[56] Rodrigo D., Jolie R., Loey A.V., Hendrickx M. (2007). Thermal and high pressure stability of tomato lipoxygenase and hydroperoxide lyase. J. Food Eng..

[57] Wang F., Du B.-L., Cui Z.-W., Xu L.-P., Li C.-Y. (2017). Effects of high hydrostatic pressure and thermal processing on bioactive compounds, antioxidant activity, and volatile profile of mulberry juice. Food Sci. Technol. Int..

[58] Oliver-Simancas R., Diaz-Maroto M.C., Perez-Coello M.S., Alanon M.E. (2020). Viability of pre-treatment drying methods on mango peel by-products to preserve flavouring active compounds for its revalorisation. J. Food Eng..

[59] Taiti C., Costa C., Menesatti P., Caparrotta S., Bazihizina N., Azzarello E., Petrucci W.A., Masi E., Giordani E. (2015). Use of volatile organic compounds and physicochemical parameters for monitoring the post-harvest ripening of imported tropical fruits. Eur. Food Res. Technol..

[60] Cheng C.-x., Jia M., Gui Y., Ma Y. (2020). Comparison of the effects of novel processing technologies and conventional thermal pasteurisation on the nutritional quality and aroma of Mandarin (*Citrus unshiu*) juice. Innov. Food Sci. Emerg. Technol..

[61] Rajkumar G., Shanmugam S., Galvao M.D., Sandes R.D.D., Neta M., Narain N., Mujumdar A.S. (2017). Comparative evaluation of physical properties and volatiles profile of cabbages subjected to hot air and freeze drying. LWT Food Sci. Technol..

[62] Rajkumar G., Shanmugam S., Galvâo M.d.S., Leite Neta M.T.S., Dutra Sandes R.D., Mujumdar A.S., Narain N. (2017). Comparative evaluation of physical properties and aroma profile of carrot slices subjected to hot air and freeze drying. Dry. Technol..

[63] Diaz-Maroto M.C., Perez-Coello M.S., Cabezudo M.D. (2002). Effect of drying method on the volatiles in bay leaf (*Laurus nobilis* L.). J. Agric. Food Chem..

[64] de Torres C., Diaz-Maroto M.C., Hermosin-Gutierrez I., Perez-Coello M.S. (2010). Effect of freeze-drying and oven-drying on volatiles and phenolics composition of grape skin. Anal. Chim. Acta.

[65] Rouseff R., Naim M. (2000). Citrus flavor stability. Flavor Chemistry.

[66] Verret C., Ballestra P., Cruz C., Pardon P., Largeteau A., El Moueffak A.H. (2008). The effect of high hydrostatic pressure on black truffle (*Tuber melanosporum*) flavour compounds. J. Phys. Conf. Ser..

[67] Mirondo R., Barringer S. (2017). Effect of peels on quality attributes of mango puree held for different times. J. Food Process. Preserv..

[68] Lalel H.J.D., Singh Z., Tan S.C. (2004). Ripening temperatures influence biosynthesis of aroma volatile compounds in ‘Kensington Pride’ mango fruit. J. Hortic. Sci. Biotechnol..

[69] Zabetakis I., Koulentianos A., Orruno E., Boyes I. (2000). The effect of high hydrostatic pressure on strawberry flavour compounds. Food Chem..

[70] Araya X.I.T., Smale N., Zabaras D., Winley E., Forde C., Stewart C.M., Mawson A.J. (2009). Sensory perception and quality attributes of high pressure processed carrots in comparison to raw, sous-vide and cooked carrots. Innov. Food Sci. Emerg. Technol..

[71] Wang R., Ding S., Zhao D., Wang Z., Wu J., Hu X. (2016). Effect of dehydration methods on antioxidant activities, phenolic contents, cyclic nucleotides, and volatiles of jujube fruits. Food Sci. Biotechnol..

[72] Song J.X., Bi J.F., Chen Q.Q., Wu X.Y., Lyu Y., Meng X.J. (2019). Assessment of sugar content, fatty acids, free amino acids, and volatile profiles in jujube fruits at different ripening stages. Food Chem..

[73] Gonzalez-Cebrino F., Garcia-Parra J., Ramirez R. (2016). Aroma profile of a red plum puree processed by high hydrostatic pressure and analysed by SPME-GC/MS. Innov. Food Sci. Emerg. Technol..

[74] Beekwilder J., Alvarez-Huerta M., Neef E., Verstappen F.W.A., Bouwmeester H.J., Aharoni A. (2004). Functional characterization of enzymes forming volatile esters from strawberry and banana. Plant Physiol..

[75] Defilippi B.G., Kader A.A., Dandekar A.M. (2005). Apple aroma: Alcohol acyltransferase, a rate limiting step for ester biosynthesis, is regulated by ethylene. Plant Sci..

[76] Qiu L., Zhang M., Bhandari B., Wang B. (2020). Effects of infrared freeze drying on volatile profile, FTIR molecular structure profile and nutritional properties of edible rose flower (*Rosa rugosaflower*). J. Sci. Food Agric..

[77] Castro J.B., Ramanathan A., Chennubhotla C.S. (2013). Categorical dimensions of human odor descriptor space revealed by non-negative matrix factorization. PLoS ONE.

[78] Liu H., An K., Su S., Yu Y., Wu J., Xiao G., Xu Y. (2020). Aromatic characterization of mangoes (*Mangifera indica* L.) using solid phase extraction coupled with gas chromatography-mass spectrometry and olfactometry and sensory analyses. Foods.

[79] Zhu J.C., Xiao Z.B. (2018). Characterization of the major odor-active compounds in dry jujube cultivars by application of gas chromatography-olfactometry and odor activity value. J. Agric. Food Chem..

[80] Wang L., Zhu J., Wang Y., Wang X., Chen F., Wang X. (2018). Characterization of aroma-impact compounds in dry jujubes (*Ziziphus jujube* Mill.) by aroma extract dilution analysis (AEDA) and gas chromatography-mass spectrometer (GC-MS). Int. J. Food Prop..

[81] Garcia-Parra J., Gonzalez-Cebrino F., Ramirez R. (2020). Volatile compounds of a pumpkin (*Cucurbita moschata*) puree processed by high pressure thermal processing. J. Sci. Food Agric..

[82] Pan X., Wu J., Zhang W., Liu J., Yang X., Liao X., Hu X., Lao F. (2021). Effects of sugar matrices on the release of key aroma compounds in fresh and high hydrostatic pressure processed Tainong mango juices. Food Chem..

[83] Zhao L., Wang S., Liu F., Dong P., Huang W., Xiong L., Liao X. (2013). Comparing the effects of high hydrostatic pressure and thermal pasteurization combined with nisin on the quality of cucumber juice drinks. Innov. Food Sci. Emerg. Technol..

[84] Baldermann S., Kato M., Kurosawa M., Kurobayashi Y., Fujita A., Fleischmann P., Watanabe N. (2010). Functional characterization of a carotenoid cleavage dioxygenase 1 and its relation to the carotenoid accumulation and volatile emission during the floral development of *Osmanthus fragrans* Lour. J. Exp. Bot..

[85] Huang F.-C., Molnar P., Schwab W. (2009). Cloning and functional characterization of carotenoid cleavage dioxygenase 4 genes. J. Exp. Bot..

[86] Bechoff A., Dhuique-Mayer C., Dornier M., Tomlins K.I., Boulanger R., Dufour D., Westby A. (2010). Relationship between the kinetics of β-carotene degradation and formation of norisoprenoids in the storage of dried sweet potato chips. Food Chem..

[87] Jeyaprakash S., Heffernan J.E., Driscoll R.H., Frank D.C. (2020). Impact of Dry. Technologies on tomato flavor composition and sensory quality. LWT Food Sci. Technol..

[88] Tandon K.S., Baldwin E.A., Scott J.W., Shewfelt R.L. (2003). Linking sensory descriptors to volatile and nonvolatile components of fresh tomato flavor. J. Food Sci..

[89] Xing Y., Lei H., Wang J., Wang Y., Wang J., Xu H. (2017). Effects of different drying methods on the total phenolic, rosmarinic acid and essential oil of purple perilla leaves. J. Essent. Oil Bear. Plants.

[90] Xu J., He Z., Zeng M., Li B., Qin F., Wang L., Wu S., Chen J. (2017). Effect of xanthan gum on the release of strawberry flavor in formulated soy beverage. Food Chem..

[91] Zhang J., Kang D., Zhang W., Lorenzo J.M. (2021). Recent advantage of interactions of protein-flavor in foods: Perspective of theoretical models, protein properties and extrinsic factors. Trends Food Sci. Technol..

[92] Rajkumar G., Rajan M., Araujo H.C., Jesus M.S., Leite Neta M.T.S., Sandes R.D.D., Narain N. (2020). Comparative evaluation of volatile profile of tomato subjected to hot air, freeze, and spray drying. Dry. Technol..

[93] Luo W., Tappi S., Wang C.F., Yu Y., Zhu S.M., Rocculi P. (2018). Study and optimization of high hydrostatic pressure (HHP) to improve mass transfer and quality characteristics of candied green plums (*Prunus mume*). J. Food Process. Preserv..

