# Supplementary material for: Effects of High Hydrostatic Pressure Combined with Vacuum-Freeze Drying on the Aroma-Active Compounds in Blended Pumpkin, Mango, and Jujube Juice
Source: Foods. 2021 Dec 20;10(12):3151. doi: 10.3390/foods10123151 (PMC8702150; doi:10.3390/foods10123151)
Supplement: Supplementary file 1 [file foods-10-03151-s001.zip › foods-1484880-supplementary.pdf]

Table S1. The result of test of HHP*VFD between-subjects effects in fresh, HHP3, VFD, and HHP3-VFD samples.

No.	Compounds	df	F	Sig	η^2
A1	Acetone	1	10.57*	0.0120	0.57
A3	Ethyl vinyl ketone	1	77.87*	< 0.0001	0.91
A4	1-(1,3-Dimethyl-3-cyclohexen-1-yl) ethanone	1	1763.70*	< 0.0001	1.00
A5	Acetoin	1	1560.96*	< 0.0001	1.00
A6	6-Methyl-5-hepten-2-one	1	1007.45*	< 0.0001	0.99
A7	4'-Methyl acetophenone	1	18824.00*	< 0.0001	1.00
A8	β-Damascenone	1	1154.55*	< 0.0001	0.99
A9	Geranylacetone	1	2.46	0.1550	0.24
A10	β-Ionone	1	7.46*	0.0260	0.48
B1	Acetaldehyde	1	19.09*	0.0020	0.71
B2	2-Methylbutyraldehyde	1	10.44*	0.0120	0.57
B3	Isovaleraldehyde	1	2.33	0.1650	0.23
B4	Valeraldehyde	1	5.13	0.0530	0.39
B5	Hexanal	1	454.97*	< 0.0001	0.98
B6	3-Methyl-2-butenal	1	102.88*	< 0.0001	0.93
B7	Heptaldehyde	1	81.89*	< 0.0001	0.91
B8	(*E*)-2-Hexenal	1	13.08*	0.0070	0.62
B9	Furfural	1	52.95*	< 0.0001	0.87
B10	(*E*)-2-Heptenal	1	32.65*	< 0.0001	0.80
B11	1-Nonanal	1	305.85*	< 0.0001	0.98
B12	5-Ethyl-1-cyclopentene-1-carboxaldehyde	1	523.20*	< 0.0001	0.99
B13	(*E*)-2-Octenal	1	591.35*	< 0.0001	0.99
B14	(*E*, *E*)-2,4-Heptadienal	1	0.06	0.8210	0.01
B15	Benzaldehyde	1	40.92*	< 0.0001	0.84
B16	(*E*)-2-Nonenal	1	0.46	0.5150	0.06
B17	(*E*, *Z*)-2,6-Nonadienal	1	144.19*	< 0.0001	0.95
B18	β-Cyclocitral	1	6.27*	0.0370	0.44
B19	5-Hydroxymethylfurfural	1	83780.95*	< 0.0001	1.00
C1	3-Carene	1	72.72*	< 0.0001	0.90
C2	α-Phellandrene	1	819.40*	< 0.0001	0.99
C3	Myrcene	1	8.46*	0.0200	0.51
C4	β-Thujene	1	73.54*	< 0.0001	0.90
C5	4-Carene	1	0.81	0.3940	0.09
C6	DL-Limonene	1	49.43*	< 0.0001	0.86
C7	Terpinolene	1	1484.3*	< 0.0001	1.00
C8	α, p-Dimethylstyrene	1	0.55	0.4790	0.06
D1	1-Penten-3-ol	1	162867.00*	< 0.0001	1.00
D2	2-Methyl-1-butanol	1	21312.00*	< 0.0001	1.00
D3	1-Pentanol	1	1.75	0.2230	0.18

D4	(*E*)-3-Hexen-1-ol	1	1.76	0.2210	0.18
D5	1-Octen-3-ol	1	102.43*	< 0.0001	0.93
D6	p-Cymen-8-ol	1	0.10	0.7580	0.01
D7	Benzyl alcohol	1	6.90*	0.0300	0.46
D8	Carveol	1	40.19*	< 0.0001	0.83
E1	Acetic acid	1	39.62*	< 0.0001	0.83
E2	Propionic acid	1	76.13*	< 0.0001	0.91
E3	Butanoic Acid	1	57.62*	< 0.0001	0.88
E4	2-Methyl butanoic acid	1	26.78*	0.0010	0.77
E5	Valeric acid	1	66.77*	< 0.0001	0.89
E6	Hexanoic acid	1	41.14*	< 0.0001	0.84
E7	Heptanoic acid	1	67.60*	< 0.0001	0.89
E8	Octanoic acid	1	34.65*	< 0.0001	0.81
E9	Benzoic acid	1	28.20*	0.0010	0.78
F1	Methyl acetate	1	5.57*	0.0460	0.41
F2	Ethyl acetate	1	18.50*	0.0030	0.70
F3	Methyl butyrate	1	316.57*	< 0.0001	0.98
F4	Ethyl butyrate	1	10.89*	0.0110	0.58
F5	Methyl valerate	1	21.72*	0.0020	0.73
F6	Isoamyl acetate	1	239.07*	< 0.0001	0.97
F7	Methyl hexanoate	1	11.27*	0.0100	0.59
F8	Methyl heptanoate	1	297.07*	< 0.0001	0.97
F9	Methyl benzoate	1	6.30*	0.0360	0.44
F10	3-Phenylpropionic acid methyl ester	1	166.71*	< 0.0001	0.95

* means significance at $p < 0.05$ level.

Table S2. Identification of aroma-active compounds in blended juice blended juices treated by HHP and VFD by DF and OAV analyses.

No.[a]	Compounds	Descriptor[b]	DF[c]						Threshold (μg/kg)[d]	OAV[e]					
			HHP-treated				HHP-VFD-treated			HHP-treated				HHP-VFD-treated	
			0 MPa (Fresh)	200 MPa (HHP1)	400 MPa (HHP2)	600 MPa (HHP3)	0 MPa (VFD)	600 MPa (HHP3-VFD)		0 MPa (Fresh)	200 MPa (HHP1)	400 MPa (HHP2)	600 MPa (HHP3)	0 MPa (VFD)	600 MPa (HHP3-VFD)
A3	Ethyl vinyl ketone	pungent	-	-	-	6	-	-	0.9[1]	-	-	-	2.51	-	-
A5	Acetoin	sweet, creamy	-	6	6	6	-	6	-	-	-	-	-	-	-
A6	6-Methyl-5-hepten-2-one	citrus, green	7	7	8	8	8	8	-	-	-	-	-	-	-
A7	4'-Methyl acetophenone	sweet	-	-	-	6	-	-	-	-	-	-	-	-	-
A10	β-Ionone	floral	-	-	-	-	6	6	0.007[2]	-	-	-	-	1333.23	1520.79
B3	Isovaleraldehyde	leafy	-	6	6	-	6	8	0.15[3]	27.35	11.23	14.80	11.75	31.42	34.55
B4	Valeraldehyde	green, grass	-	-	7	6	-	-	12[2]	1.30	-	1.62	1.32	-	-
B5	Hexanal	fruity, grass	6	7	7	8	8	8	4.5[2]	4.95	3.94	17.31	22.80	5.14	1.96
B8	(*E*)-2-Hexenal	green, fatty	7	6	6	7	8	6	17[3]	23.15	8.59	10.31	8.74	5.09	3.32
B11	1-Nonanal	orange peel	-	-	6	7	8	-	1[3]	-	-	1.89	3.30	1.96	-
B13	(*E*)-2-Octenal	fresh, waxy	-	-	8	7	8	8	3[2]	-	-	2.73	2.50	2.98	2.36
B15	Benzaldehyde	nutty, bitter almond	7	6	-	-	-	-	-	-	-	-	-	-	-
B16	(*E*)-2-Nonenal	fatty, green	6	7	7	-	6	-	0.5[3]	35.33	5.40	7.45	7.60	14.74	-
B17	(*E, Z*)-2,6-Nonadienal	cucumber	8	-	-	-	-	8	0.02[2]	1076.69	-	-	91.87	421.36	279.04
B18	β-Cyclocitral	tropical, herbal	-	-	-	-	7	6	5[4]	-	-	-	-	1.56	1.43
C1	3-Carene	citrus, pine	-	-	-	-	6	-	-	-	-	-	-	-	-
D3	1-Pentanol	oil, sweet	-	-	-	6	-	-	-	-	-	-	-	-	-

D4	(*E*)-3-Hexen-1-ol	green, earthy	6	-	8	7	8	-	-	-	-	-	-	-	-
D5	1-Octen-3-ol	earthy, oily	8	8	8	8	8	8	1[3]	5.15	7.43	8.33	10.89	17.07	10.43
E2	Propionic acid	pungent	8	8	8	8	-	-	-	-	-	-	-	-	-
E3	Butanoic acid	sharp, cheese	6	-	8	-	-	-	-	-	-	-	-	-	-
E4	2-Methyl butanoic acid	pungent, cheese	8	8	8	8	-	-	-	-	-	-	-	-	-
E5	Valeric acid	sweaty, rancid	-	6	-	-	-	-	-	-	-	-	-	-	-
E6	Hexanoic acid	sour, sweaty	8	7	8	8	8	6	-	-	-	-	-	-	-
E7	Heptanoic acid	fruity, sweat	6	-	-	-	-	-	-	-	-	-	-	-	-
E8	Octanoic acid	waxy, vegetable	-	6	7	-	8	-	-	-	-	-	-	-	-
F1	Methyl acetate	-	-	-	-	-	-	-	2[5]	2.42	-	-	-	-	-
F2	Ethyl acetate	-	-	-	-	-	-	-	8.5[6]	4.73	1.25	-	-	-	1.22
F3	Methyl butyrate	fruity, sweet	-	-	-	6	-	-	15.1[6]	4.78	1.29	-	-	-	-
F4	Ethyl butyrate	-	-	-	-	-	-	-	1.1[6]	88.79	13.00	4.99	3.58	-	-
F6	Isoamyl acetate	-	-	-	-	-	-	-	2[2]	2.36	-	-	-	-	-
F9	Methyl benzoate	-	-	-	-	-	-	-	0.52[2]	18.79	10.37	14.83	11.50	12.05	10.09

[a] Number assignment referred to Table 2.
[b] Odor description as perceived at the sniffing port; '-' means not detected by assessors.
[c] DF, total times detected by 4 assessors at 8 trials; '-' means the volatiles were detected by assessors less than 6 times..
[d] Odor thresholds in water taken from the references; '-' indicates the volatile concentrations are lower than their threshold value.
[e] OAV values are given as means (n = 3); '-' indicates OAV is lower than 1.

References:

1. Ahmed, E.M.; Dennison, R.A.; Dougherty, R.H.; Shaw, P.E. Flavor and odor thresholds in water of selected orange juice com-ponents. J. Agric. Food Chem. 1978, 26, 187-191, doi:10.1021/jf60215a074.
2. Pino, J.A.; Mesa, J. Contribution of volatile compounds to mango (Mangifera indica L.) aroma. Flavour Fragr. J. 2006, 21, 207-213, doi:10.1002/ffj.1703.
3. Buttery, R.G.; Ling, L.C. Additional studies on flavor components of corn tortilla chips. J. Agric. Food Chem. 1998, 46, 2764-2769, doi:10.1021/jf980125b.
4. Buttery, R. Quantitative and sensory aspects of flavor of tomato and other vegetables and fruits. Flavor science: Sensible prin-ciples and techniques. 1993, 259-286.
5. Balavoine, P. Observations Sur Les qualités Olfactives Et Gustatives Des Aliments. Mitteilungen aus dem Gebiete der Lebens-mittel-untersuchung un Hygiene Travaux de chimie alimentaire et d'hygiene 1948, 39, 342-350.
6. Pino, J.; Torricella, R.; Orsi, F. Correlation between sensory and gas-chromatographic measurements on grapefruit juice vola-tiles. Nahrung-Food 1986, 30, 783-790.
